# Synthesis, Characterization, and Biological Studies of a Piperidinyl Appended Dipicolylamine Ligand and Its Rhenium Tricarbonyl Complex as Potential Therapeutic Agents for Human Breast Cancer

**DOI:** 10.1155/2016/2675937

**Published:** 2016-10-25

**Authors:** Amali Subasinghe, Inoka C. Perera, Svetlana Pakhomova, Theshini Perera

**Affiliations:** ^1^Department of Chemistry, University of Sri Jayewardenepura, Nugegoda, Sri Lanka; ^2^Department of Zoology and Environmental Science, University of Colombo, Colombo, Sri Lanka; ^3^Department of Chemistry, Louisiana State University, Baton Rouge, LA, USA

## Abstract

A novel ligand bearing a central piperidinyl sulfonamide group, N(SO_2_pip)dpa, and its corresponding Re tricarbonyl complex, [Re(CO)_3_(N(SO_2_pip)dpa)]^+^, have been synthesized in good yield. The methylene CH_2_ signal seen as a singlet (4.54 ppm) in a ^1^H NMR spectrum of the ligand in DMSO-*d*_6_ appears as two doublets (5.39, 5.01 ppm) in a spectrum of the [Re(CO)_3_(N(SO_2_pip)dpa)]^+^ complex and confirms the presence of magnetically nonequivalent protons upon coordination to Re. Structural results revealed that the Re–N bond lengths fall within the normal range establishing coordination of ligand to metal. The presence of intraligand *π* → *π* ^⁎^ and *n* → *π* ^⁎^ transitions is indicated by the absorption peaks around 200–250 nm in UV-visible spectra. Absorption peaks in UV-visible spectra around 300 nm for metal complexes were identified as MLCT transitions. The S–N stretch observed as a strong peak at 923 cm^−1^ for N(SO_2_pip)dpa appeared at a shorter frequency, at 830 cm^−1^ in an FTIR spectrum of the [Re(CO)_3_(N(SO_2_pip)dpa)]^+^. The intense fluorescence displayed by the N(SO_2_pip)dpa ligand has quenched upon coordination to Re. Relatively low IC_50_ values given by human breast cancer cells, MCF-7, (N(SO_2_pip)dpa = 139 *μ*M, [Re(CO)_3_(N(SO_2_pip)dpa)]^+^ = 360 *μ*M) indicate that N(SO_2_pip)dpa and [Re(CO)_3_(N(SO_2_pip)dpa)]^+^ are promising novel compounds that can be further investigated on their usage as potential anticancer agents.

## 1. Introduction

Historically,* fac*-Re(CO)_3_L complexes have provided a good model system to interpret the nature of its analogous ^99m^Tc agents which are widely used in imaging. The* fac*-[Re(CO)_3_(H_2_O)_3_]^+^ precursor may be easily utilized [1] for the synthesis of new organometallic complexes having suitable pharmaceutical properties. Furthermore, the triaquatricarbonyl complex of ^99m^Tc, which is the group seven congener of rhenium, is commercially available as a kit preparation [2], enabling the easy simulation of the nonradioactive rhenium complexes [3]. The use of nonradioactive rhenium facilitates the synthesis of the metal complexes rather than handling radioactive metal tracers.

Metal complexes (M = ^99m^Tc, Re) bearing symmetrical linear tridentate ligands have been reported to possess lower bioaccumulation than complexes bearing bidentate or monodentate ligands [2], resulting in fewer side effects when used on the long term. Rhenium, in its lowest oxidation state, is less sensitive to chemical attack by the reactive species that may be present in cellular environment [2]. Facially arranged carbonyl ligands cover one face of the distorted octahedral complex thus protecting the metal center. Metal carbon bonds are greatly stabilized by strong backdonation from the carbonyl ligand to the metal. As rhenium is a soft metal center, it prefers soft donors such as nitrogen and tridentate metal complexes with nitrogen donors are widely used [2, 4, 5].

Among various types of chronic human diseases, certain types of cancers are widely treated by organometallic complexes [6, 7]. Breast cancer is one of the most abundant types of cancer in recent times. However, only a few metallopharmaceuticals are available for diagnostic and therapeutic purposes for human breast cancer [7, 8].

Sigma receptors are a distinct class of proteins that are mostly found in the central nervous system [9]. Sigma receptors are virtually absent in healthy human breast cells; however, they appear in a high density in breast tumor biopsy tissue [9, 10]. Proliferative human breast cancer cells express approximately ten times more sigma receptors per cell than the quiescent cells [9]. Therefore, high densities of sigma receptors in rapidly proliferating breast cancer cells make them ideal candidates for diagnosis as well as therapy of breast cancer. Due to the multiple binding sites containing lipophilic sterol binding domains on the protein molecule, various types of exogenous ligands, such as several derivatives of piperidine, piperazine [11, 12], benzamides [13], and alkylamine [14], have been reported as potential pharmaceuticals for therapy of human breast cancer, mainly because these types of compounds preferentially bind with sigma receptors [10].

To the best of our knowledge, there are only a few radiotracers in which the piperidinyl group has been utilized towards targeting sigma receptors; Satpati et al. have synthesized ^99m^Tc based cancer diagnostic agents [10], Choi et al. have developed a ^99m^Tc labeled sigma-2 receptor-specific ligand as a potential breast tumor imaging agent [9], and Caveliers et al. have utilized* N*-[2-(1′-piperidinyl)ethyl]-3-^123^I-iodo-4-methoxybenzamide [8]. Only the latter has entered clinical trials for the diagnosis of patients with primary breast cancer.

We based our study on the fact that* fac*-[Re(CO)_3_(N(R)dpa)]^+^ complexes have been reported to possess promising biomedical properties where R equals a suitable substituent linked by a N–C bond at the central nitrogen in dipicolylamine [15]. Furthermore, the tertiary sulfonamide linkage has recently been utilized to propose a new approach to radiopharmaceutical bioconjugation [5]. We also envisage that the target specificity may be increased by incorporating a piperidinyl derivative into the novel compounds.

**Scheme 1:**
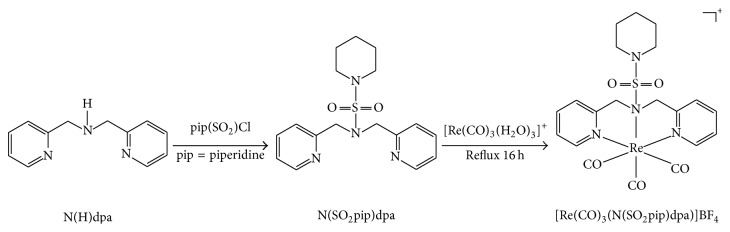
Synthetic routes for the preparation of N(SO_2_pip)dpa and [Re(CO)_3_(N(SO_2_pip)dpa)]BF_4_.

Our goal has been to synthesize a novel ligand and its rhenium tricarbonyl complex having possible therapeutic potential, with the long term goal of extending this study to ^99m^Tc complexes towards the eventual diagnosis of breast cancer. Thus, in this study we report the synthesis and characterization of a novel piperidinyl appended dipicolylamine ligand and its corresponding Re tricarbonyl complex and go one step further to report their potential applicability in biological systems as anticancer agents (Scheme 1).

## 2. Experimental

### 2.1. Materials and Methods

Re_2_(CO)_10_, AgOTf, piperidine-1-sulfonyl chloride, di(2-picolyl)amine, NaBF_4_, anhydrous sodium sulphate, acetone, methanol, dichloromethane, chromasolv water, and dioxane were obtained from Sigma Aldrich, USA. Human breast cancer cell line MCF-7 was obtained from American Type Culture Collection. All the solvents and chemicals were of analytical grade and were used as received, without further purification.

### 2.2. NMR Measurements

^1^H NMR spectra were recorded in DMSO-*d*_6_ on a Bruker 400 MHz spectrometer. Peak positions are relative to trimethylsilane (TMS) as reference. All NMR data were processed with TopSpin 3.2 and MestReC software.

### 2.3. X-Ray Data Collection and Structure Determination

Single crystals were placed in a cooled nitrogen gas stream at 90 K on a Bruker Kappa Apex-II DUO diffractometer equipped with Cu* Kα* (*λ* = 1.54178 Ǻ) radiation (N(SO_2_pip)dpa ligand) or Mo* Kα* radiation (*λ* = 0.71073 Å) ([Re(CO)_3_(N(SO_2_pip)dpa)]BF_4_ complex). Refinement was performed by full-matrix least squares methods using SHELXL (Sheldrick (2008)) [16], with H atoms in idealized positions. Molecular graphics are drawn using* ORTEP-3* for windows [17].

### 2.4. UV-Visible Spectroscopy

Electronic spectra for ligand and metal complex were obtained on Spectro UV-VIS autoversion 3.10, UV-2602 spectrophotometer. The spectral range was 190 nm–1100 nm. Spectra were obtained in methanol with base line correction. Spectral data were processed with UV WIN software.

### 2.5. FTIR Analysis

FTIR spectra were recorded on a Thermo Scientific Nicolet iS10 spectrophotometer. ATR spectra were obtained within the 4000–600 cm^−1^ spectral range. Spectral data were processed with OMNIC software.

### 2.6. Fluorometric Analysis

Excitation and emission spectra for ligand and metal complex were obtained in methanol and acetonitrile on a Thermo Scientific Lumina spectrophotometer. A 150 W Xenon lamp was used as the excitation source. Spectral data were processed with Luminous software.

### 2.7. Synthesis

In order to synthesize metal complexes, [Re(CO)_3_(H_2_O)_3_]OTf precursor was prepared using Re_2_(CO)_10_ as the starting material according to a known procedure [1].

#### 2.7.1. N(SO_2_pip)dpa Ligand

A solution of piperidine-1-sulfonyl chloride (0.034 g, 5 mmol) in 25 mL of dioxane was added dropwise over a period of 2 hours to a solution of N(H)dpa (0.057 g, 10 mmol) in 100 mL of dioxane at 20°C. The reaction mixture was stirred at room temperature for 24 hours and then filtered to remove any precipitate. Thereafter, the dioxane was completely removed by rotary evaporation. Slightly acidic water (30 mL, pH ~ 5) was added to the resulting compound, and the product was extracted into CH_2_Cl_2_ (2 × 25 mL). The CH_2_Cl_2_ extracts were combined, washed with water (2 × 25 mL), and taken to dryness. Grey color, plate-like crystals were obtained. ^1^H NMR signals (ppm) in DMSO-*d*_6_ are 8.49(d, 2H, H6/H6′), 7.75(t, 2H, H4/H4′), 7.35(d, 2H, H3/H3′), 7.27(t, 2H, H5/H5′), 4.53(s, 2CH_2_), 3.09(s, 2H, Ha), and 1.40(s, 2H, Hb).

#### 2.7.2. [Re(CO)_3_(N(SO_2_pip)dpa)]BF_4_ Complex

A solution of N(SO_2_pip)dpa (0.0346 g, 0.1 mmol) in 3 mL methanol was treated with aqueous [Re(CO)_3_(H_2_O)_3_]OTf (0.1 mmol, 4 mL). Methanol (3 mL) was added to dissolve the precipitate which formed. Acidity of the solution was measured (pH ~ 6) and the clear solution was heated at reflux for 16 h. A slight excess of NaBF_4_ was added to the clear, brownish yellow color solution. The solution was then allowed to develop crystals over three days at room temperature after which block-like, colorless crystals were obtained. ^1^H NMR signals (ppm) in DMSO-*d*_6_ are 8.84(d, 2H, H6/H6′), 8.06(t, 2H, H4/H4′), 7.61(d, 2H, H3/H3′), 7.47(t, 2H, H5/H5′), 5.39(d, endo-H), 5.01(d, exo-H), 3.68(s, 2H, Ha), and 1.68(s, 2H, Hb).

### 2.8. Biological Assays

#### 2.8.1. Cytotoxicity Assessment

The novel ligand and its metal complex were investigated for their cytotoxicity against MCF-7 (breast cancer) cells. Cells were cultured in 96-well culture plates and exposed to 25 *μ*g/mL, 50 *μ*g/mL, 100 *μ*g/mL, 200 *μ*g/mL, and 400 *μ*g/mL concentrations of ligand and complex for 24 h, 48 h, and 72 h, respectively, and cytotoxicity was assessed by Sulforhodamine B assay [18]. All exposures were carried out in triplicate. Briefly, the cell supernatant was completely removed and washed with phosphate buffer solution. Trichloroacetic acid (50%, 25 *μ*L) was added on top of fetal bovine serum-free fresh medium (200 *μ*L) to make final concentration of 10% trichloroacetic acid and was incubated at 4°C for one hour former to the SRB assay. The plate was then washed with five washing cycles with water and dried completely. An aliquot of 100 *μ*L of 0.4% Sulforhodamine B dissolved in 1% trichloroacetic acid, was added to each well, and was allowed to stain for 15 minutes. The plate was again washed with five washing cycles to remove unbound dye using 1% (vol/vol) acetic acid after removing the stain. The protein bound dye was solubilized with trisbase (10 mM, pH 7.5, 200 *μ*L), after air-drying. The plates were then shaken for 60 minutes to homogenize the dye solution. The absorbance was then measured at 540 nm using Synergy* HT*BioTek microplate reader. The percentage viability was calculated by the equation given below:

(1) Viable  cell%=Absorbance  of  treated  cellsAbsorbance  of  untreated  cells×100.

## 3. Results and Discussion

### 3.1. Structural Results

Crystal data and details of the structural refinement for N(SO_2_pip)dpa and [Re(CO)_3_(N(SO_2_pip)dpa)]^+^ are summarized in Table 1. Crystallographic data are deposited with the Cambridge Crystallographic Data Centre under deposition numbers CCDC 1495607 and 1495608.

**Figure 1:**
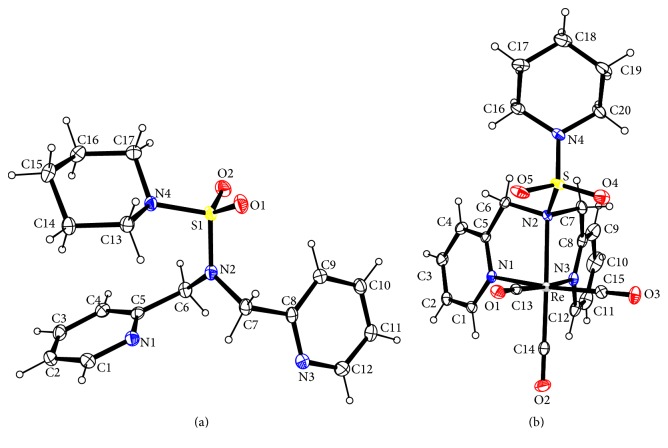
ORTEP plot of N(SO_2_pip)dpa (a) and the cation in [Re(CO)_3_(N(SO_2_pip)dpa)]^+^ (b). Thermal ellipsoids are drawn with 50% probability.

The uncoordinated ligand (Figure 1) possesses S–N(2) bond length of 1.6194 (11) Å (Table 2). However, upon forming the metal complex, the S–N(2) bond length has increased (1.777 (2) Å). This may be attributed to the donation of lone electron pair on sulfonamide nitrogen to Re, which results in lowering of the strength of the S–N bond. Previous studies have reported that the S–N distance is ~1.73–1.76 Å [5] when Re–N(sulfonamide) bond is strong [19]. The above observation provides evidence that the complexation of the ligand with Re has occurred successfully. Weakly coordinated or noncoordinated sulfonamide groups have considerable double character. This is because of the conjugation of lone electron pair of the sulfonamide nitrogen with S=O across the S–N bond [19].

In the N(SO_2_pip)dpa ligand, planar geometry is observed at N2 nitrogen which is sp^2^ hybridized. Therefore, C6, C7, and S species are oriented in an angle of ~120° to the central N2 (Table 3). All the other bond angles of the ligand lie within the normal values depending on their hybridization.

In the [Re(CO)_3_(N(SO_2_pip)dpa)]^+^ complex, the coordination geometry at Re exhibits a pseudo octahedral structure (Figure 1). This deviation may be due to the facial arrangement of tridentate chelating unit which leads to forming two strained, five-membered rings at Re resulting in a N–Re–N bond angle < 90°. The remaining three coordination sites of the opposite face of the octahedron are occupied by three carbonyl ligands.

The distance of new bonds formed between Re and nitrogen atoms in dipicolylamine lies within the range of normal values. Generally, the distance between Re and sp^2^ hybridized nitrogen in a Re complex with prototypical NNN donor ligand is 2.14–2.18 Å [4, 20]. The coordination bond between Re and sp^2^ hybridized nitrogen in the pyridyl rings which are denoted as N1 and N3 (Figure 1) exhibits a bond distance close to that of the prototypical bond (Table 4). Similarly, the coordination bond between Re and sp^3^ hybridized tertiary nitrogen (2.251 (2) Å), though exceeding the normal range (2.23–2.29 Å) [4, 20], is less than the Re–N2 distance of 2.2826 (16) Å of [Re(CO)_3_(N(SO_2_Me)dpa]PF_6_ [5] and confirms rehybridization of sulfonamide nitrogen from sp^2^ to sp^3^ when bound to Re. Further evidence for the rehybridization of sulfonamide nitrogen from sp^2^ to sp^3^ upon binding to Re is provided by the fact that the bond angles of this tertiary sulfonamide nitrogen are closer to 109.5° (Table 5). All the other bond angles lie within the normal range of reported compounds [5].

### 3.2. ^1^H NMR Analysis

**Figure 2:**
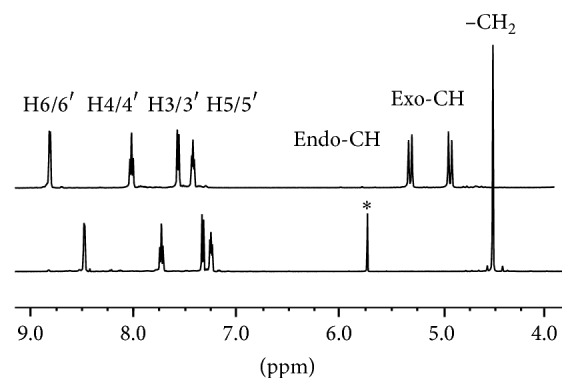
^1^H NMR spectra in DMSO-*d*_6_ of N(SO_2_pip)dpa (lower) and [Re(CO)_3_(N(SO_2_pip)dpa)]^+^ (upper). ^*∗*^Peak due to trace amounts of CH_2_Cl_2_ solvent.

N(SO_2_pip)dpa and [Re(CO)_3_(N(SO_2_pip)dpa)]^+^ were characterized using ^1^H NMR spectroscopy in DMSO-*d*_6_. Peaks related to residual solvents were also identified [21, 22]. Signals related to the dipicolylamine were assigned according to previously reported data of related compounds [5, 23]. Protons attached to the pyridyl nitrogen (H6/6′) are more deshielded and thereby signals are located downfield in the spectrum (Figure 2). Protons in the piperidinyl ring were assigned with the aid of reported spectra of related compounds [10]. The signal for protons in the methylene groups of the N(SO_2_pip)dpa appears as a singlet at (4.53 ppm) because the protons attached to the two methylene groups are magnetically equivalent due to free rotation. Singlet peaks at 3.3, 2.5, 5.8, and 3.5 ppm are due to residual solvents including water, DMSO, dichloromethane, and dioxane, respectively [21, 22].

According to the spatial arrangement of the atoms in [Re(CO)_3_(N(SO_2_pip)dpa)]^+^, the methylene protons are projected towards and away from the carbonyl ligands attached to rhenium and may be designated as* endo*-H and* exo*-H, respectively. Therefore, those methylene protons are not magnetically equivalent. Hence, the singlet at 4.53 ppm related to the CH_2_ group of the free ligand appears as two doublets further downfield upon binding to the metal (Figure 2). All the signals related to protons attached to pyridyl nitrogen have shifted downfield upon binding to the metal (Table 6). These shifts of peaks may be attributed to the electron withdrawing inductive effect as a result of the formation of direct Re–N bond and provide strong evidence of the formation of the metal complex.

### 3.3. UV-Visible Analysis

**Figure 3:**
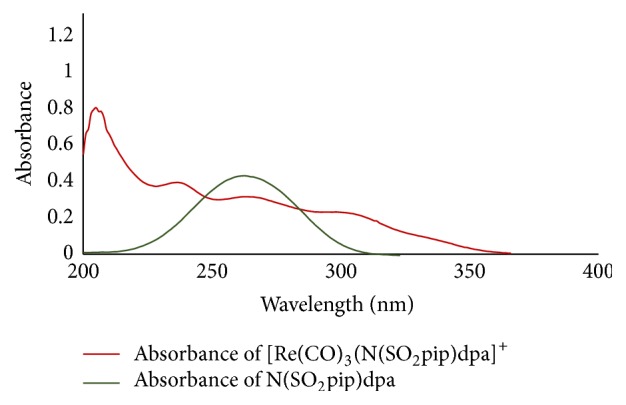
UV-visible spectra of N(SO_2_pip)dpa and [Re(CO)_3_(N(SO_2_pip)dpa)]^+^.

The absorption peak at 307 nm in [Re(CO)_3_(N(SO_2_pip)dpa)]^+^ may be attributed to MLCT transition (Figure 3). Rhenium tricarbonyl complexes with suitable ligands display MLCT transitions [24]. Other high energy absorption peaks may be due to intraligand *π* → *π* ^*∗*^ and *n* → *π* ^*∗*^ transitions [24, 25].

### 3.4. FTIR Analysis

Literature has greatly aided in assigning FTIR spectra of novel ligand and complex. In an FTIR spectrum of N(SO_2_pip)dpa, a short absorption band appears at 3077 cm^−1^ due to the asymmetric stretching vibration of aliphatic systems [26]. A narrow and sharp absorption band at 2942 cm^−1^ is attributed to the C–H asymmetric stretching vibration of aliphatic systems [26]. A strong absorption band at 1136 cm^−1^ may be due to the symmetric stretching vibration of S=O bond of the sulfonamide group [26]. A collection of absorption peaks in the range between 1294 cm^−1^ and 1590 cm^−1^ is due to the symmetric and asymmetric stretching vibrations of C=C bonds [26] in aromatic rings and C=N stretching vibration mode in the ligand [26]. Stretching vibration due to S–N bond is identified as the strong absorption peak at 923 cm^−1^ [27].

Most of the peaks attributed to ligand also appear in a spectrum of the [Re(CO)_3_(N(SO_2_pip)dpa)]^+^ complex and, in addition, two strong and intense absorption peaks at 2035 cm^−1^ and 1916 cm^−1^ are attributed to the stretching vibrations of CO ligands in the Re(CO)_3_ core [28]. C–N stretching vibrations in the pyridyl rings have shifted towards lower frequencies. S–N stretching vibration has shifted to a lower frequency (836 cm^−1^) due to lowering of initial bond energy resulting from the sigma donation of lone electron pairs on sp^3^ hybridized orbital in sulfonamide nitrogen.

### 3.5. Fluorometric Analysis

Fluorescence spectra were obtained for N(SO_2_pip)dpa and [Re(CO)_3_(N(SO_2_pip)dpa)]^+^ in methanol and acetonitrile. The concentrations of the test samples were approximately 0.01 mol/dm^3^. 3D scans were done for the analysis and the relevant excitation and emission details are summarized in Table 7.

**Figure 4:**
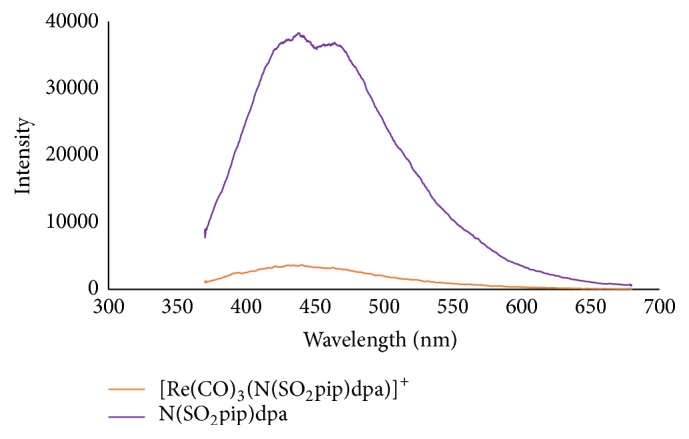
Fluorescence spectra of N(SO_2_pip)dpa and [Re(CO)_3_(N(SO_2_pip)dpa)]^+^ in acetonitrile.

N(SO_2_pip)dpa displays high fluorescence emission in methanol and the intensity was enhanced even more in acetonitrile (Figure 4). These emissions may occur due to the intraligand *π* → *π* ^*∗*^ and *n* → *π* ^*∗*^ transitions. However, fluorescence intensity of [Re(CO)_3_(N(SO_2_pip)dpa)]^+^ has lowered possibly due to the quenching of fluorescence upon direct binding of sulfonamide nitrogen to the metal. Nevertheless, weak fluorescence of [Re(CO)_3_(N(SO_2_pip)dpa)]^+^ may arise due to the MLCT transition.

### 3.6. Antiproliferative Activity

**Figure 5:**
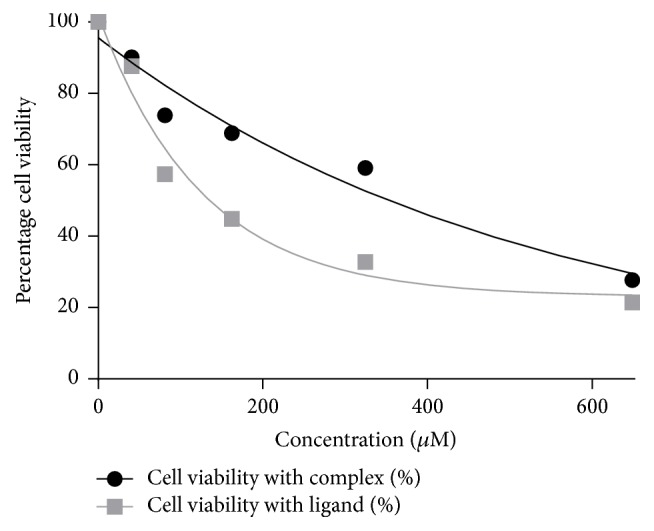
The plot of percentage cell viability versus concentration of the test compounds (*μ*M) obtained by Sulforhodamine B assay.

Cytotoxicity in human breast cancer cells (MCF-7) was induced upon addition of ligand and complex at IC_50_ of 139 *μ*M and 360 *μ*M, respectively, after 24 hr (Figure 5). This shows that, comparatively, human breast cancer cells show higher sensitivity to the ligand than its metal complex. These findings emphasize that N(SO_2_pip)dpa and [Re(CO)_3_(N(SO_2_pip)dpa)]^+^ are promising novel compounds that can be further investigated on their usage as anticancer agents and cancer cell imaging agents.

Comparisons of the IC_50_ values obtained for ligand and the complex at 24, 48, and 72 hr have shown that the minimum test compound concentration needed to affect cell viability increased with increasing incubation time (Table 8) for the complex whereas it has decreased with that of the ligand. This emphasizes that, comparatively, ligand cytotoxicity is much higher than the metal complex toxicity. Decreasing cytotoxicity may be an indicator of activation of repair mechanisms after the initial cellular damage.

**Figure 6:**
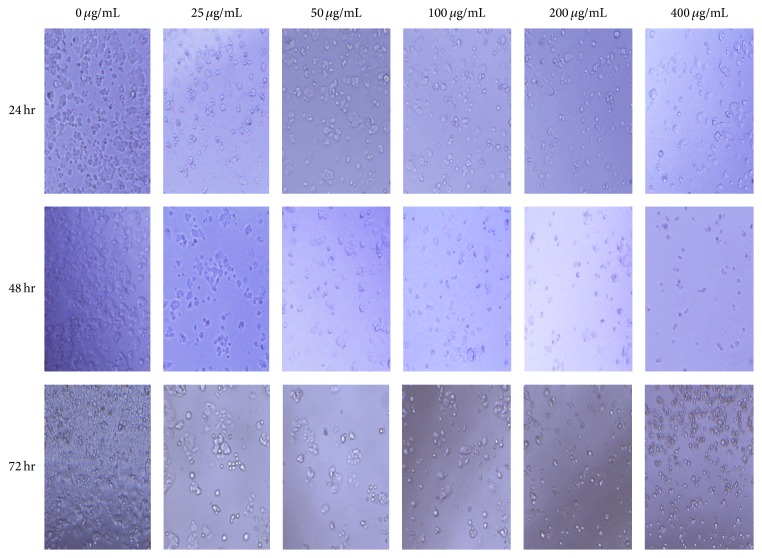
Morphology of human breast cancer cells (MCF-7), after 24, 48, and 72 hr incubating with the N(SO_2_pip)dpa ligand at increasing concentrations.

**Figure 7:**
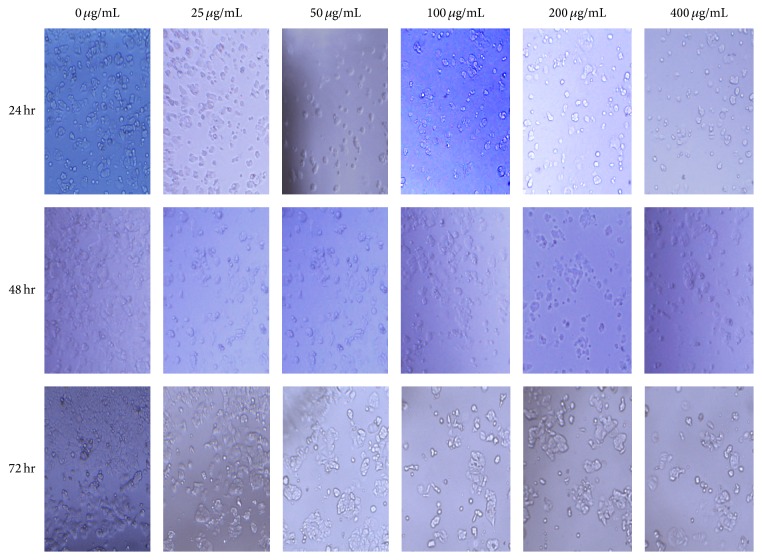
Morphology of human breast cancer cells (MCF-7), after 24, 48, and 72 hr incubating with the [Re(CO)_3_(N(SO_2_pip)dpa)]^+^ complex at increasing concentrations.

Morphology of human breast cancer cells upon treating with test compounds at 24, 48, and 72 hr time periods was micrographed and images were obtained as in Figures 6 and 7.

Morphology of the cells does not show significant change under light microscopy indicative of antiproliferative activity. However, the cell numbers in wells have an apparent change. Beside cytotoxic effects, the ligand shows an acute effect by 48 hours but numbers seem to be increasing by 72 hours. Cells may have resorted to a repair mechanism against the cytotoxic effect of the ligand. However, when complexed with Re, the decline of cell population seems to be continuing beyond 72 hr, which shows a persistent effect and continued antiproliferative activity.

## 4. Conclusions

A novel ligand and its corresponding rhenium complex were synthesized in good yield and purity and characterized using single crystal X-ray diffraction, NMR, UV-visible, and FTIR spectroscopy. The near-normal Re–N bond lengths as well as the relatively long N–S bond demonstrate that tertiary sulfonamide serves as a good donor in the [Re(CO)_3_(N(SO_2_pip)dpa)]^+^ complex. The sp^2^ to sp^3^ rehybridization of sulfonamide nitrogen has facilitated the binding of tertiary sulfonamide nitrogen in the [Re(CO)_3_(N(SO_2_pip)dpa)]^+^ complex. The fluorescence of the free N(SO_2_pip)dpa ligand was quenched upon direct binding of the sulfonamide nitrogen to Re in the metal complex resulting in weak fluorescence for [Re(CO)_3_(N(SO_2_pip)dpa)]^+^.

Cytotoxicity for both N(SO_2_pip)dpa and [Re(CO)_3_(N(SO_2_pip)dpa)]^+^ was determined against human breast cancer cells (MCF-7) by Sulforhodamine B assay. Effects of complex on breast cancer cells seem to decrease with time but the ligand continues to exert the cytotoxic effect where both compounds show promising antiproliferative activity.

In conclusion, utilizing the conjugation approach described by Perera and coworkers [5], we demonstrate that a piperidinyl group may be successfully introduced to develop therapeutic and diagnostic agents because we envisage that the synthesis reported here for nonradioactive rhenium complexes may be extended to the ^99m^Tc core.

## Figures and Tables

**Table 1:** Crystal data and structure refinement for N(SO_2_pip)dpa and [Re(CO)_3_(N(SO_2_pip)dpa)]^+^.

Crystal data	N(SO_2_pip)dpa	[Re(CO)_3_(N(SO_2_pip)dpa)]^+^
Empirical formula	C_17_H_22_N_4_O_2_S	C_20_H_22_N_4_O_5_ReS·BF_4_0.682(CH_3_OH)·0.318(H_2_O)
*M*r	346.44	731.03
Crystal system	Triclinic	Monoclinic
Space group	$P\overset{-}{1}$	*P*2_1_/*c*
Unit cell dimensions		
*a* (Å)	8.6557 (3)	10.8932 (4)
*b* (Ǻ)	10.1132 (3)	11.7517 (5)
*c* (Ǻ)	10.4022 (3)	20.7973 (8)
*α* (deg)	71.979 (1)	—
*β* (deg)	81.262 (1)	102.260 (2)
*γ* (deg)	74.382 (1)	—
*V* (Å^3^)	831.59 (5)	2601.62 (18)
*T* (K)	90	90
*Z *	2	4
*ρ*_calc_ (g/cm^3^)	1.384	1.866
abs coeff (mm^−1^)	1.88	4.82
2*θ*_max_ (deg)	135	66.2
*R*[*F* ^2^ > 2*σ*(*F* ^2^)]	0.030	0.027
*wR*(*F*2)	0.086	0.058
Res density	0.29, −0.41	1.50, −1.65
Data/parameters	2819/217	9917/354
Radiation type	Cu *Kα*	Mo *Kα*
Radiation wavelength/(Å)	1.54178	0.71073

**Table 2:** Selected bond distances (Å) for N(SO_2_pip)dpa.

	Bond length (Å)
S1–O1	1.4363 (10)
S1–O2	1.4369 (10)
S1–N2	1.6194 (11)
S1–N4	1.6329 (11)
N1–C5	1.3391 (18)
N1–C1	1.3422 (18)
N3–C8	1.3373 (18)
N3–C12	1.3459 (18)
N2–C7	1.4657 (16)
N2–C6	1.4615 (16)
N4–C13	1.4848 (16)
N4–C17	1.4838 (17)

**Table 3:** Selected bond angles (deg) for N(SO_2_pip)dpa.

	Bond angle (deg)
O1–S1–O2	116.88 (6)
O1–S1–N2	106.19 (6)
O2–S1–N2	111.30 (6)
O1–S1–N4	113.83 (6)
O2–S1–N4	106.15 (6)
N2–S1–N4	101.48 (5)
N2–C6–C5	113.65 (10)
N1–C5–C4	122.65 (12)
N1–C5–C6	116.86 (11)
C4–C5–C6	120.47 (12)
C5–N1–C1	117.42 (11)
C8–N3–C12	117.28 (11)
C6–N2–C7	118.01 (10)
C6–N2–S1	118.33 (9)
C7–N2–S1	122.54 (9)
C17–N4–C13	113.03 (10)

**Table 4:** Selected bond distances (Ǻ) for [Re(CO)_3_(N(SO_2_pip)dpa)]^+^.

	Bond length (Ǻ)
Re–N1	2.1746 (19)
Re–N2	2.251 (2)
Re–N3	2.179 (2)
Re–C13	1.919 (3)
Re–C14	1.904 (3)
Re–C15	1.934 (2)
S–O4	1.425 (2)
S–O5	1.422 (2)
S–N2	1.777 (2)
S–N4	1.588 (2)

**Table 5:** Selected bond angles (deg) for [Re(CO)_3_(N(SO_2_pip)dpa)]^+^.

	Bond angle (deg)
N1–Re–N3	77.55 (7)
N1–Re–N2	77.98 (8)
N3–Re–N2	76.58 (8)
C13–Re–C15	88.25 (10)
C13–Re–N1	98.09 (9)
N2–Re–C15	97.00 (9)
N2–Re–C14	171.70 (9)
N2–Re–C13	98.43 (9)
C14–Re–N3	96.37 (10)
C15–Re–N3	95.81 (9)
C6–N2–Re	108.74 (14)
C7–N2–Re	107.06 (15)
S–N2–Re	113.31 (9)
N1–Re–C15	172.39 (9)
N1–Re–C14	96.36 (9)

**Table 6:** Comparison of the ^1^H NMR shifts of N(SO_2_pip)dpa ligand and [Re(CO)_3_(N(SO_2_pip)dpa)]^+^ complex in DMSO-*d*_6_.

	H6/6′	H5/5′	H4/4′	H3/3′	−CH_2_
N(SO_2_pip)dpa	8.49 (d)	7.27 (t)	7.75 (t)	7.35 (d)	4.53 (s)
[Re(CO)_3_(N(SO_2_pip)dpa)]^+^	8.84 (d)	7.47 (t)	8.06 (t)	7.61 (d)	5.39 (d), 5.01 (d)

**Table 7:** Excitation and emission wave lengths of N(SO_2_pip)dpa and [Re(CO)_3_(N(SO_2_pip)dpa)]^+^ in methanol and acetonitrile.

Test sample	Solvent	Excitation wave length/nm	Emission wave length/nm
N(SO_2_pip)dpa	Methanol	320	468
	Acetonitrile	325	475

[Re(CO)_3_(N(SO_2_pip)dpa)]^+^	Methanol	320	430
	Acetonitrile	325	435

**Table 8:** IC_50_ values reported for ligand and the complex at 24, 48, and 72 hr incubation period.

Test compound	IC_50_ values/*µ*M		
	24 hr	48 hr	72 hr
Ligand	262	209	95
Complex	283	391	915
